# Addressing workplace gender inequality: Using the evidence to avoid common pitfalls

**DOI:** 10.1111/bjso.12606

**Published:** 2022-11-22

**Authors:** Michelle K. Ryan

**Affiliations:** ^1^ Global Institute for Women's Leadership The Australian National University Canberra Australian Capital Territory Australia; ^2^ Faculty of Business and Economics University of Groningen Groningen The Netherlands

**Keywords:** gender differences, gender equality, glass cliff, intersectionality, risk‐taking

## Abstract

In this Landmark article I outline four common missteps that are made when designing and implementing workplace gender equality initiatives: (1) when we don't go beyond describing the numbers; (2) when we try to ‘fix’ women rather than fix systems; (3) when we are overly optimistic about the progress we have made; and (4) when we fail to recognise the intersectionality of the experiences that women face. I will briefly consider each of these missteps in term, presenting research that suggests alternative ways of approaching gender equality initiatives.

## INTRODUCTION

Despite much progress in the past 50 years, workplace gender inequality remains a persistent problem. Worldwide, women only occupy about 37 per cent of leadership roles (World Economic Forum, 2022), the pay gap sits at approximately 20 per cent (International Labour Oragnisation, 2022), and women remain concentrated in low‐status, low‐paid jobs (UN Women, 2022). There are countless initiatives designed to address workplace gender equality—those that try to attract women to certain professions and roles where they are under‐represented, those that try to support women's career trajectories, and the those that try to retain women in the workforce. While the impetus behind these initiatives is generally positive, many of these interventions are not based on evidence, in terms of their design, their implementation or in the evaluation of their efficacy.

Most infamous in this space are those initiatives that build on an understanding that much gender discrimination (but certainly not all) is a result unconscious bias. The research most cited to underpin unconscious bias training is work on implicit prejudice and implicit associations (e.g. Devine, 1989; Greenwald et al., 1998; Greenwald & Banaji, 1995). While there has been theoretical, methodological and psychometric debate about the utility of implicit tests such as the IAT (e.g. Blanton & Jaccard, 2006; Nosek & Sriram, 2007; see also Jost, 2019) what is of more interest here is the utility of unconscious bias training itself. While unconscious bias training is good at awareness raising, it is less effectual at achieving behaviour change or increased gender equality (e.g. Atewologun et al., 2018; Bezrukova et al., 2016; Kalev et al., 2006) and has been shown to have unintended negative consequences such as backfiring or feelings of false progress (e.g. Dover et al., 2020; Leslie, 2019).

In my current role, as the Director of the Global Institute for Women's Leadership at The Australian National University, I have three key responsibilities (1) to conduct research to better understand gender inequality, (2) to work with organizations and government to translate the evidence base into effective policy and practice and (3) to advocate for social change and gender equality. It is at the nexus of these three endeavours that I can see where we get it right, and where we, unfortunately, get it wrong.

In this Landmark article I outline four common missteps that are made when designing and implementing workplace gender equality initiatives: (1) when we do not go beyond describing the numbers; (2) when we try to ‘fix’ women rather than fix systems; (3) when we are overly optimistic about the progress we have made; and (4) when we fail to recognize the intersectionality of the experiences that women face. I will briefly consider each of these missteps in term, presenting research that suggests alternative ways of approaching gender equality initiatives.^1^

## WHEN NUMBERS JUST AREN'T ENOUGH

One of the first steps in many gender equality action plans is to do an audit of the representation of women. How many women are in the organization? How many women are in decision‐making roles? How many women are there in senior management and on the boards of directors? This number crunching extends to describing other inequalities: How big is the gender pay gap? How many women were promoted in the last promotions round? What is the success rate of female job applicants? This approach is common in many internal organizational gender equality plans (Ely & Thomas, 2020), and as part of many external accreditation programmes (e.g. Rosser et al., 2019). Understanding representation and understanding key metrics of gender equality are a necessary part of achieving gender equality—but they are not sufficient. Such numbers are a great starting point as they identify problem areas to be rectified. But they do not tell the whole story.

In this section, I will outline a body of research on women in leadership and the glass cliff (Haslam & Ryan, 2008; Ryan & Haslam, 2005, 2007) that illustrates why we cannot just stop at descriptive numbers. This work suggests that it is not enough to know *whether* women are in leadership positions, but *when* they are in leadership positions. It also illustrates the importance of looking at women's experiences in such positions. And finally, it illustrates the importance of understanding the psychological processes behind the appointment of women to leadership positions.

This body of research builds on the metaphor of the glass ceiling, that describes the under‐representation of women in leadership positions, to examine the conditions under which women are likely to be appointed to leadership positions. Almost 20 years of research has demonstrated the phenomenon whereby women are more likely to be appointed to leadership roles during times of crisis (see Morgenroth et al., 2020, & Ryan et al., 2016, for meta‐analyses and an overview). With the extension of the glass ceiling metaphor—the glass cliff—we hoped to capture the riskiness and precarity of such leadership positions: to give a sense of occupying a position up on high, yet of teetering on the edge.

The phenomenon of the glass cliff was first uncovered as a reaction to a newspaper article in *The Times* (Judge, 2003). This article presented evidence that companies that had more women on their boards of directors, had poorer share prices, and thus the increasing number of women on UK corporate board was ‘wreaking havoc’ on corporate Britain (p. 21). In response, Ryan and Haslam (2005) proposed an alternative analysis, whereby rather than women causing poor company performance, it was poor company performance that led to women being appointed to boards of directors. We conducted nuanced analysis of board appointments and monthly changes in company share prices that showed that this alternative explanation was indeed the case—(the small number of) women who were appointed to boards of directs, were appointed after a prolonged period of poor share price performance. Share price afterwards did not differ from their male counterparts.

Since this first discovery of the phenomenon, a global body of research on the glass cliff has emerged, one that uses multiple methodologies (archival analyses, experimental studies, case studies, qualitative work) to demonstrate the nuance and underlying processes associated with the glass cliff phenomenon (Morgenroth et al., 2020; Ryan et al., 2016). The glass cliff is not restricted to corporate settings, and has also been found in (a) the political sphere (e.g. Kulich et al., 2015; Ryan et al., 2010)—as illustrated by all three of the UK's female Prime ministers: Thatcher (1980s recession), May (Brexit) and Truss (energy crisis and spiralling inflation); (b) sporting contexts (e.g. Wicker et al., 2019); and (c) in non‐government, third sector organizations (e.g., Bogacz‐Wojtanowska et al., 2018).

The importance of the glass cliff here is that it points to the necessity of looking beyond simply the number of women in leadership positions, to understand the circumstances under which women are likely to be appointment to such positions. If we just take the proportion of women in leadership roles as a measure of gender equality, then glass cliff appointments may be seen as an example of progress towards gender equality. But in reality, the opposite may be the case.

The context in which the glass cliff occurs can lead to such positions representing a new and subtle form of sexism or gender discrimination. Such a poisoned chalice potentially sets women up for additional scrutiny, stress and risk of failure. Indeed, the very risk and precarity experienced by those in glass cliff positions may hinder progress towards gender equality. Women in glass cliff positions are likely to face greater challenges in their leadership roles, such as (a) being blamed for negative conditions that were set in train long before they were appointed (Ryan & Haslam, 2005), shorter tenure (Glass & Cook, 2016) or (c) stress and burnout (Ryan et al., 2009). These additional difficulties may contribute to the stagnation of women's representation in leadership positions, reinforcing stereotypes that women are not suited to leadership.

The glass cliff is just one example where the complexity of gender equality might be hidden behind the top‐line numbers. Understanding the subtlety and nuance behind the numbers gives us a truer sense of our progress towards gender equality. We can think of these in terms of who, when, why and where questions. For example, *who* bears the brunt of gender inequality—we know that gender inequality is fundamentally intersectional, being exacerbated by other group memberships (see Section ‘When we are overly optimistic’, below). *When* and *where* does inequality occur. And the big question for us as psychologists, is the *why*—what are the processes sitting behind the numbers, what drives inequality, and in turn, what do we need to do to help mitigate it.

Exploring beyond the numbers can also help inform us of the most effective ways to attack those problems. In the case of the glass cliff, looking beyond the number of appointments raises a whole new set of research questions to be asked (and answered). Are women preferentially selected by others for leadership in times crisis (yes, according to Haslam & Ryan, 2008)? Are women appointed because we think they are good at dealing with crisis (no, according to Kulich et al., 2015; Ryan et al., 2011). Do women select these positions because they like a challenge (also no, according to Rink et al., 2012)?

## WHEN WE TRY TO FIX WOMEN

The question of whether women self‐select into glass cliff positions leads us nicely into our next misstep—the tendency to focus on women when trying to solve the problem of gender inequality. Many of the approaches to improving gender equality recognize that the issues arise from inequalities embedded in our social and organizational structures and systems. Key here are the traditional gender stereotypes about what women and men *are* like (Ellemers, 2018) and what they *should be* like (Heilman, 2012). In particular, many workplace inequalities arise because the societal view of women's warmth is incompatible with societal views of leadership and success that prioritize notions of agency and competence (e.g. Koenig et al., 2011; Schein, 1973). Importantly our social and organizational structures and systems are predicated on these gender norms and stereotypes (Eagly et al., 2000), including recruitment, promotion and reward practices; parental leave and childcare policies; and educational systems.

However, this acknowledgement of systemic basis of gender equality often dissipates when it comes to actually implementing interventions and initiatives. There is a relatively consistent underlying assumption within these initiatives that gender inequalities can be addressed with a focus on individual competencies. From this perspective, we can narrow the gender equality gaps by providing women with additional skills and training. For example, initiatives to encourage girls and women in science, technology, engineering and maths (STEM) are often focused on boosting their engagement and ambition (Liben & Coyle, 2014). Leadership training courses often focus on teaching women ‘girl boss’ leadership skills (Atir, 2022) and encouraging them to take greater risks and make bigger sacrifices, overcome impostor symptom, be authentic at work and negotiate the next promotion or pay rise (Hackworth et al., 2018). This approach is epitomized by the ‘lean in’ approach to gender equality (Sandberg, 2013), which seeks to encourage women to make the right choices and have the right mindset.

All of these approaches have, as their implicit theory of change, an understanding that women are in some way broken and not up to the task. The solution is, therefore, seen to be to ‘fix’ them—to change their behaviours, address their skills deficit, remedy their mindset. But the evidence is very clear on this point—it is not women that need fixing, but the deeply entrenched systems of gender inequality that structure our organizations and structure society more broadly.

Below I outline some illustrative research that demonstrates that women's engagement and belonging, their feelings of impostor syndrome and their willingness to take risks are not individual‐level problems that renders them needing to be fixed. Rather, these issues are a direct product of organizational and societal systems, and their experiences in these systems and thus require structural solutions.

### Engagement and belonging

One area in which this approach is highly visible is trying to attract and retain girls and women in male‐dominated sectors, such as STEM, finance and construction. Many of initiatives designed to increase gender inequality in these spaces focus on trying to increase girls' and women's interest for and engagement with these sectors (McKinnon, 2022), such as the heavily criticized campaign—Science: It's a Girl Thing—from the European Commission, which featured women in fashionable PPE making lipstick (Grosu, 2013). What is implicit here is that there is some sort of inherent lack of enthusiasm in women, that needs to be addressed, rather than the fact that women and girls are responding to very real cultural and normative barriers that exclude them (Saucerman & Vasquez, 2014).

In a series of studies looking at women in surgery—where women make up less than 25% per cent of the profession—Peters et al. (2012) examined whether the under‐representation of women may be explained, at least in part, by women's perceptions of, and experiences within, the profession. Across two studies we demonstrated that female surgical trainees perceived a lack of fit between themselves and the prototypical masculine surgeon. In turn, this perceived lack of fit was associated with a reduction in identification with the profession and an increased desire to opt out of the profession.

Similarly, work by Meeussen et al. (2022) demonstrate than in male‐dominated careers, such as surgery and the veterinary profession, women (compared to men) report less career engagement because of their more frequent experiences of gender discrimination and lower perceived fit with those higher up the career ladder. In turn, these barriers predicted reduced expectations of success in their field and expected success of their sacrifices, which in turn predicted lower willingness to make sacrifices.

Together, these studies suggest the role that external barriers, such as experiences of discrimination and perceptions of fit, play in women's career decision making in male‐dominated professions. Thus, trying to attract and retain women in these spaces by focusing on women themselves is unlikely to be fruitful. Rather, interventions need to address the root of the problem, discriminatory environments and a lack of role models if they want women to come and women to stay (see Casad et al., 2018).

### Imposter syndrome

Another area in which has received a lot of attention when it comes to women in the workplace are initiatives that seek to address impostor syndrome. This concept is used to describe individuals who express doubts about their self‐worth, failing to take credit for their successes or attributing their successes to luck. Such individuals worry that others will see them as impostors or frauds. The very use of the term ‘syndrome’ suggests that this experience is an individual‐level problem—a condition that requires diagnosis and treatment and fixing. And indeed, there will be no surprise to find out that there are many initiatives out there that are designed to help individuals, and in particular women, to overcome ‘their’ impostor syndrome. For example, such interventions seek to increase women's confidence, reduce their perfectionism and change their mindsets (Chandra et al., 2019).

However, as Feenstra et al. (2020) argue, rather than being seen as a personal problem that plagues individual women, it is critical to acknowledge the role that the social and organizational context plays in eliciting feelings of impostorism (see also Kark et al., 2022). Indeed, a series of studies by Begeny et al. (2022) demonstrate that impostor feelings can be seen as is a direct response to how one is treated by others. In a longitudinal study, we showed that that experiencing fewer expressions of distinctive treatment, such as being asked for advice, resulted in a significant increase in impostor feelings over time. Moreover, in experimental studies we showed that when individuals experience positive distinctive treatment from work colleagues, this significantly reduces impostor feelings.

In this way, characterizing impostor feelings at an individual level is unlikely to be useful, both in terms of running the risk of pathologizing these feelings and in terms of understanding where they come from. Thinking of impostor feelings as a context‐dependent outcome of workplace experiences has clear implications for how we ‘treat’ impostor syndrome. Rather than putting the onus on employees, particularly women, to overcome their own impostor feelings—being more confident and ‘faking it until you make it’—we need to implement more systemic approaches, creating cultures where colleagues are valued and treated with respect.

### Risk taking

One common explanation for the persistence of workplace gender inequalities is that women are less willing to take career‐enhancing risks, such as asking for a pay rise or taking on a new position (Byrnes et al., 1999). Indeed, women's risk aversion is a persistent aspect of gender stereotypes, with many arguing that this is an innate difference aspect of gender (Bem, 1974). Such an analysis has a number of issues, including the assumption that risk taking is inherent desirable and necessarily career enhancing, and because it fails to recognize the types of risks that women do take in everyday life (Morgenroth et al., 2018). But nonetheless, a key facet of the lean in approach to fixing women is encouraging women to take more risks, including memetic advice such as ‘if you are offered a seat on a rocket ship, do not ask what seat, just get on’ and ‘fortune does favour the bold, and you never know what you are capable of if you do not try’ (Sandberg, 2013).

However, research demonstrates that far from being innate, women's willingness to take risks is dependent of their experiences in the workplace. Research conducted by Morgenroth et al. (2022) looks at gender differences in risk taking through a lens of the anticipated and experienced consequences of risk taking. Across three studies, there was no evidence for gender differences in *initial* risk taking or in the anticipation of consequences for the risks with which women and men had no prior experience. However, when we looked at actual experiences of risk taking in the workplace—such as taking on a difficult task, speaking up or quitting your job for a new job—men reported more positive consequences for taking risks than women, and as a result, anticipated having a greater likelihood of taking the same risks in the future.

Studies like this question the assumption that it is women's innate risk aversion that underlies workplace gender inequalities. Rather they demonstrate that any aversions women have are likely to be a consequence of their workplace experiences, and indeed, are likely to be informed by the gendered, negative experiences they have when attempting to take risks. For this reason, gender equality initiatives that focus on encouraging women to take more risks are unlikely to succeed, and it is the gendered costs and benefits for risk that need to be addressed.

Taken together, this exploration of some of the common ways in which initiatives target gender equality issues—engagement, impostor syndrome and risk taking—suggest that framing these as individual‐level problems is unlikely to be fruitful. At best, such an approach may provide those individual women who are targeted by such initiatives, usually women that hold a certain amount of privilege (see Section 4) with a short‐term advantage. At worst, such attempts to fix women reinforce the stereotypes and norms that form the basis of structural gender inequalities and become yet another demand on women's time. Interventions should, instead, target the foundational causes of inequality: organizational systems and culture.

## WHEN WE ARE OVERLY OPTIMISTIC

If we compare where we are now on the workplace gender equality front, compared to where we have been historically, it is clear that there have been many positive changes—better gender representation, safer working conditions and more equality in terms of pay. But such changes are not linear, and neither are they inevitable. Indeed, over more recent time periods we have seen stagnation in these advances, in in some cases even backsliding (Word Economic Forus, 2022). Indeed, current forecasts suggest it will be at anywhere between 132 (Word Economic Forus, 2022) and 300 (UN Women, 2022) years before we reach global gender equality.

Part of the tension here lies in the degree to which we recognize and celebrate our gender equality accomplishments, and to what extent are we realistic about how much we still have to achieve. This decision is not just about whether or not one wants to be an optimistic person. An understanding of the degree to which gender inequalities persist, and in particular the denial of gender inequality, forms a key aspect of sexist attitudes, such as those captured by the modern sexism scale (Swim et al., 1995). Indeed, there are a number of very real consequences of failing to acknowledge the persistence of gender inequality.

Begeny et al. (2020) looked at what happens when traditionally male‐occupied professions, such as the veterinary profession, attract more women. While having a greater representation of women is clearly progress, some may take it as an indication that the discrimination is no longer a problem. We demonstrated that despite women being the majority of veterinary students and junior vets, female vets still report experiencing discrimination. In a follow‐up experimental study, we illustrated one way in which this discrimination manifests itself. Vets with managerial responsibilities evaluated a male vet as more competent and suggested paying him 8 per cent more than an equally qualified female vet. Key here, these discriminatory evaluations were evident primarily among those who believed women no longer face discrimination in the profession. Thus, even when positive change occurs, discrimination persists, ironically perpetuated by those who believe it is no longer a problem.

Research also demonstrates that progress towards gender equality may be hampered by those who overestimate the rate of progress. A study by Begeny et al. (2022) surveyed doctors in the United Kingdom who were asked to estimate the representation of women across a number of roles in the medical profession. Both male and female doctors consistently overestimated the number of women in medicine. However, while those women who over‐estimated female representation still supported gender‐equality initiatives, such as initiatives run by the Royal College of Surgeons and the General Medical Council, those men who were over‐optimistic about progress showed significantly lower levels of support. Thus, men who overestimated progress towards gender equality were at highest risk of undermining it (see also Coffé & Reiser, 2021).

Recognizing and celebrating progress towards gender equality is important for a sense of hope and collective efficacy, both necessary for continued motivation for change (e.g. Cohen‐Chen & Van Zomeren, 2018; Van Zomeren, 2013). However, studies like these suggest that there is potentially a fine line between optimism and a failure to recognize persistent inequalities. If we are to close the gap, and it would be nice if we could do so in less than a century, we need a healthy dose of realism and we need to acknowledge what still remains to be done.

## WHEN WE AREN'T INTERSECTIONAL

A final common misstep that is taken when trying to address gender inequalities is to treat women as if they are a monolithic, homogenous group. There is often a ‘one size fits all’ approach to interventions and change (Tzanakou, 2019). But the experiences within women—between individuals and between different groups of women—are often more varied than the experiences between women and men. There is a need to understand this variety in women's experiences, and how this is determined by other intersecting identities, especially those that are marginalized or stigmatized (e.g. Crenshaw, 1991).

What is most troublesome about the one size fits all approach, is that gender interventions and initiatives are most often based on the experiences of the dominant group—such as those women who are white, middle‐class or straight. This is problematic, both because the experiences of such women are by no means universal, and because women not included in this group—for example culturally and linguistically diverse women, working‐class women, and LGBTQI+ women and gender diverse people, often face the greatest inequalities.

For example, research by Opara et al. (2020) identified that Black and minoritized women's workplaces experienced were very much influenced by their racial identities, including having stereotypes and expectations imposed upon them. Indeed, research demonstrates that Black women are treated on the basis of negative stereotypes that question their competence and their legitimacy (e.g. Williams & Dempsey, 2014) or see them as aggressive and masculine (Hall et al., 2019). In contrast, Asian women may be affected by the model minority myth (Cheng et al., 2017) and be seen as hyper‐competent (Liang & Peters‐Hawkins, 2017), but at the same time face stereotypes of low agency (Ghavami & Peplau, 2013) and hyper‐femininity (Mukkamala & Suyemoto, 2018).

These differential experiences mean that homogenous workplace gender equality initiatives are unlikely to be effective. Indeed, Wong et al. (2022) argue that diversity interventions tend not to take into account the wide variety of women's experiences. Across three studies we demonstrate that women who are racially marginalized need different things from their diversity interventions than do White women. More specifically we found that while White women focused on the needs of initiatives address issues of women's agency, Black women overtly reported the need for initiatives to take into account intersectional differences, such as racialized gender stereotypes where Black women are seen as pushy or overly assertive. Similarly, Asian women reported the need to address challenges to their authority which stem from racialized stereotypes of Asian women as passive and submissive. Importantly, our textual analysis of gender equality websites showed that organizations were less likely to represent the needs of Black and Asian women—a form of intersectional invisibility (Purdie‐Vaughns & Eibach, 2008)—such that their gender equality advocacy tended to focus on (White women's) issues of agency, rather than issues of racialized stereotyping reported by Black and Asian women.

These findings suggest that if gender equality initiatives are going to be successful, they must take into account the wide variety of women's experiences and needs. Catering for just one group of women is unhelpful, particularly if that group of women as a whole are likely to experience less disadvantage. Interventions need to overtly address the issues faced by all women, not just those in the majority or those with the most privilege. This points to the importance of understanding the intersectional nature of gender inequality—taking into account that these inequalities are exacerbated and qualified by multiple forms of oppression, such as those based on race, ethnicity, class, sexuality, disability, age and linguistic diversity.

## CONCLUSIONS

While the majority of gender equality initiatives are founded on good intentions, this in and of itself is not enough to bring about significant and lasting change. As we have seen above, interventions need to be based on a clear evidence base, one that (1) looks beyond the top line numbers to the complexity and nuance of gender inequality; (2) aims to fix the things that actually needs fixing (systems and structures) rather than trying to fix women; (3) celebrates change while at the same time being realistic about the challenges that are to come; and (4) understands the inherently intersectional nature of gender inequality.

The good news is that social psychology is perfectly situated to rise to all of these challenges. First, we are well placed to understand the processes and contexts that sit behind the top line numbers. For example, as we have seen, social psychological theories can help us understanding the gendered stereotypes than underlie our social and organizational policies and practices (e.g. Eagly et al., 2000; Ellemers, 2018; Heilman, 2012; Koenig et al., 2011). They can also help us understanding how workplace experiences can affect gendered workplace choices (e.g. Begeny et al., 2022; Meeussen et al., 2022; Morgenroth et al., 2022).

Second, within our theories we have the ability to ensure we are asking the appropriate questions and that we are framing our questions at the right level of analysis—at the level of the individual, the group or at a societal level—and an understanding that the individual level is not always the most appropriate. For example, the social identity approach (Tajfel & Turner, 1979; Turner et al., 1987) provides a clear framework to examine how our group memberships, and the contexts in which we are embedded, may impact upon our attitudes and behaviours, particularly at work (Haslam, 2004).

Third, through concepts like modern sexism (Swim et al., 1995), social psychology can provide an understanding of the perniciousness of the denial of sexism and the subsequent outcomes, such as continued gender discrimination and a lack of support for gender equality initiatives (see Begeny et al., 2020, 2022). This is particularly important as such views provide a strong basis to the backlash that is levelled against gender equality initiatives (Flood et al., 2021). Indeed, more recent theories of sexism, such as the belief in sexism shift (Zehnter et al., 2021), indicate that there are increasingly prevalent views that men are now the key victims of sexism (Ryan & Zehnter, 2022), a view that is likely to exacerbate resistance to change.

Finally, while not yet an integrated part of social psychology, there are some excellent examples of how to make our research more intersectional (Bowleg, 2017; Cole, 2009; Rosenthal, 2016). This intersectionality can be implemented in terms of the types of research questions we ask and the make‐up of our samples (Purdie‐Vaughns & Eibach, 2008; Remedios & Snyder, 2015) and even the way we do open science (Sabik et al., 2021). Importantly, while much of the intersectional advances have been made at the intersection of gender and race; there is still much to be done in acknowledging other intersectional identities, such as those based on age, class, disability and sexuality.

Taken together, while the evidence shows us that there have clearly been missteps on the way, the evidence also demonstrates that social psychology is in an excellent position to play an important role as we stride forward towards gender equality.

## CONFLICTS OF INTEREST

All authors declare no conflict of interest.

## Footnotes

^1^
This Landmark Article builds on a short opinion piece I wrote for Nature: Ryan (2022)
